# Characteristics of Objective Sleep and Its Related Risk Factors Among Parkinson's Disease Patients With and Without Restless Legs Syndrome

**DOI:** 10.3389/fneur.2021.644385

**Published:** 2021-06-11

**Authors:** Shuyu Sun, Xianchao Zhao, Jiafeng Ren, Jinxiang Cheng, Junying Zhou, Changjun Su

**Affiliations:** ^1^Department of Neurology, The Second Affiliated Hospital of Fourth Military Medical University, Xi'an, China; ^2^Sleep Medicine Center, West China Hospital, Sichuan University, Chengdu, China; ^3^Department of Neurology, West China Hospital, Sichuan University, Chengdu, China

**Keywords:** Parkinson's disease, restless legs syndrome, polysomnography, periodic limb movements in sleep, objective sleep quality

## Abstract

**Objective:** This study aimed to investigate the objective sleep characteristics and their related risk factors among Parkinson's disease (PD) patients with and without restless legs syndrome (RLS).

**Methods:** A total of 125 patients with PD who underwent overnight polysomnography (PSG) were recruited consecutively. Eighty-one patients, including 27 PD with RLS (PD-RLS) and 54 PD without RLS (PD-NRLS), were included in the final analysis after 1:2 propensity score matching. Demographic, clinical, and polysomnographic data were compared between PD patients with and without RLS. The risk factors for sleep quality were examined using a multiple linear regression model.

**Results:** The prevalence of RLS among PD patients was 28.0% (35/125). The PD-RLS group exhibited a higher score for the Unified Parkinson Disease Rating Scale (UPDRS) III than the PD-NRLS group. Also, the PD-RLS patients displayed significantly shorter total sleep times, worse sleep quality, decreased stage 3 duration, a longer wake time after sleep onset, and a higher arousal index than those without RLS (all *p* < 0.05). In the multiple linear regression model, PD duration (β = −0.363, 95% CI: −0.652 to −0.074; *p* = 0.016), UPDRS-III (β = −0.356, 95% CI: −0.641 to −0.071; *p* = 0.016), and periodic limb movement index (PLMI) (β = −0.472, 95% CI: −0.757 to −0.187; *p* = 0.002) were determined to be the risk factors influencing sleep quality in PD-RLS patients. The UPDRS-III (β = −0.347, 95% CI: −0.590 to −0.104; *p* = 0.006) and HAMD scores (β = −0.343, 95% CI: −0.586 to −0.100; *p* = 0.007) were significantly associated with sleep quality after adjusting for confounding factors in PD-NRLS patients, respectively.

**Conclusions:** PD-RLS patients exhibited more disturbed and fragmented sleep in objective sleep architecture than PD-NRLS patients. The severity of motor symptoms in PD was significantly associated with poor sleep quality in both PD-RLS and PD-NRLS patients. Notably, our findings indicated that periodic limb movements during sleep (PLMS) was the risk factor that influenced the objective sleep quality in PD patients with RLS.

## Introduction

Parkinson's disease (PD) is the second most common neurodegenerative disease among people older than 65 years old in China (1). It is a movement disorder characterized by motor symptoms such as a resting tremor, rigidity, bradykinesia, and postural instability. Currently, clinicians have become increasingly aware of the management of non-motor symptoms of PD due to their significant impact on the quality of life of patients (2). Sleep disturbances appear to be the most frequent non-motor symptoms, as they are observed in up to 90% of PD patients (3, 4). The categories of sleep disturbances in patients with PD comprise insomnia, daytime sleepiness, restless legs syndrome (RLS), rapid eye movement sleep behavior disorder (RBD), and so on. The regulation of sleep and wakefulness is affected by the dysfunction of multiple brain areas and neurotransmitters in patients with PD (5).

RLS is a sleep disorder characterized by a series of sensorimotor symptoms and an irresistible urge to move the limbs. These symptoms are usually accompanied by uncomfortable and unpleasant sensations in the legs. Moreover, these symptoms start or worsen during periods of rest or inactivity and are relieved by movement (6). RLS is also a common sleep disturbance affecting a considerable number of PD patients. The global prevalence of RLS in PD patients was about 14–16% (7), and a more recent review indicated that the prevalence of RLS in PD patients could be as high as 52.3% (5), which was mostly higher than the prevalence of 1.9–4.6% in the general population (8). A longitudinal study reported that the prevalence of RLS increased from 4.6 to 16.3% during the progression course of PD (9). Notably, both disorders respond well to dopaminergic replacement therapy (10, 11) and share an association with periodic leg movements during sleep (12), which strongly suggest commonalities in the pathogenesis association between PD and RLS. Thus, far, it is not clear whether RLS is a manifestation of the early pathological process of PD or occurs with the progression of PD. Therefore, the hypothesis of dysfunction of the central dopaminergic system in RLS should be verified in the development of RLS in PD in the future study.

It has been reported that PD patients with RLS (PD-RLS) experience more severe sleep problems, exhibiting a significantly higher Pittsburgh Sleep Quality Index (PSQI) score and lower Parkinson's Disease Sleep Scale (PDSS) score than PD patients without RLS (PD-NRLS) (13, 14). Recently, one study indicated that PD-RLS might be related to more severe Parkinsonism, depression, cognitive dysfunction, poor sleep quality, and a worse quality of life (15–17). However, considering that the diagnosis of RLS is usually based on clinical symptoms, few studies of sleep quality in PD-RLS are included in objective assessments. In the current study, we evaluated the objective sleep quality and explored the potential influencing factors on sleep among PD patients with or without RLS.

## Materials and Methods

### Study Design and Participants

This retrospective observational study was performed between January 2015 and January 2020 at the Sleep Medicine Center and the Neurology Department of the Tangdu Hospital of the Fourth Military Medical University. This study was a part of a cross-sectional study that examined sleep disorders of patients with PD. The study was conducted under the Declaration of Helsinki and was approved by the Local Ethics Committee and Institutional Review Board of the Tangdu Hospital of the Fourth Military Medical University.

**Figure 1:**
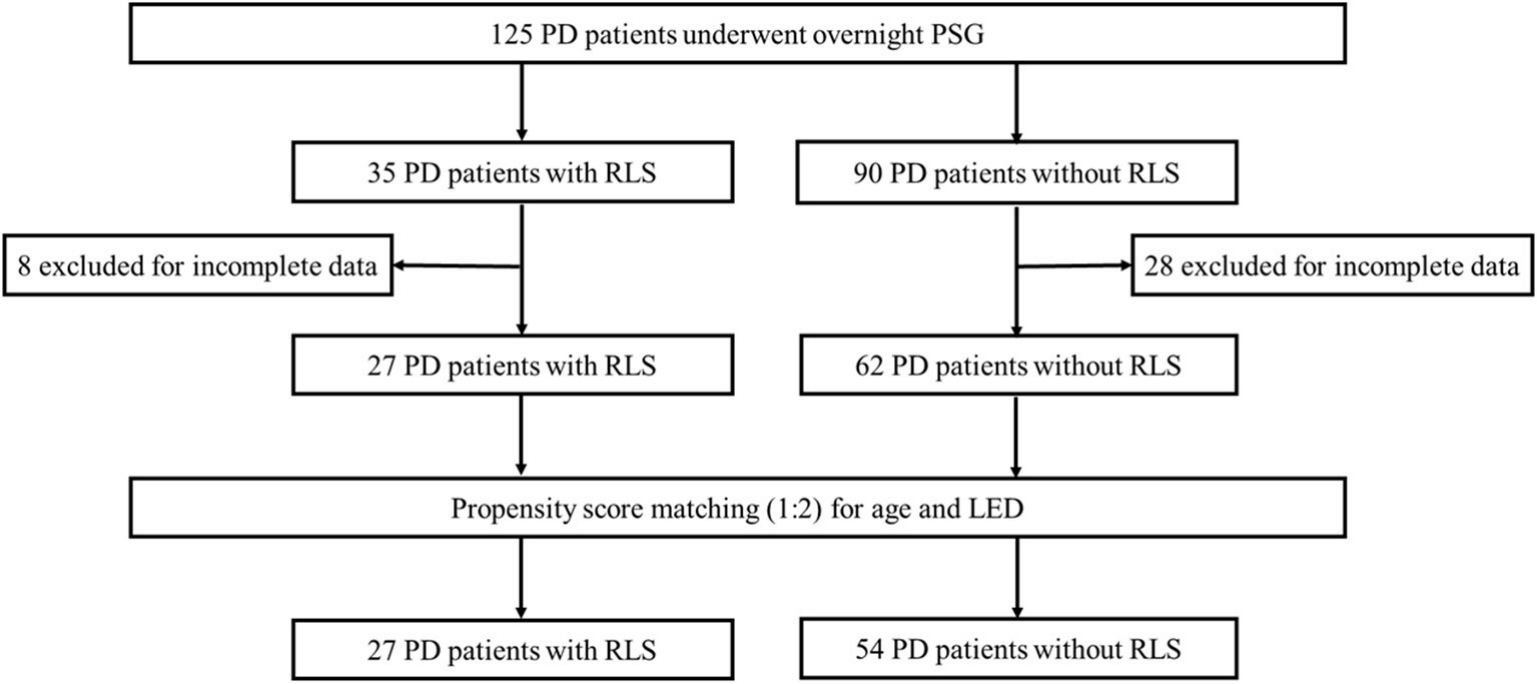
Flow chart diagram of the selection of PD patients included in the analyses. PD, Parkinson's disease; PSG, polysomnography; RLS, restless legs syndrome; LED, levodopa equivalent dose.

In this cross-sectional study, all the hospitalized patients with PD were consecutively included and received PSG assessments regardless of any complaints of sleep disorders. A total of 125 PD patients were recruited into the study. Thirty-six patients were excluded because of incomplete clinical information or polysomnography (PSG) data. After screening, 27 PD patients were identified to exhibit RLS, and 62 PD patients did not have RLS. To reduce selection bias and potential baseline confounding factors, propensity score matching (PSM) was used to adjust the baseline clinical characteristics. Age and the levodopa equivalent dose (LED) were included in the PSM model to assess their possible influences on sleep quality. The body mass index (BMI) was used for balance calculations. PD patients with and without RLS were matched 1:2 without replacement using a nearest-neighbor approach and caliper restrictions (0.4). Finally, 27 PD patients with RLS (PD-RLS group) and 54 PD patients without RLS (PD-NRLS group) were included in the statistical analyses (Figure 1).

### Clinical Evaluation

Demographic characteristics, including age, age at onset of PD, gender, and BMI, were collected. Disease duration and medication history were reviewed from the patients' medical records. The diagnosis of PD was based on Movement Disorder Society clinical diagnostic criteria for PD (18). The motor symptoms and the PD stage were evaluated using the Unified Parkinson Disease Rating Scale (UPDRS) III and the Modified Hoehn and Yahr Scale, respectively (19). RLS was confirmed according to the 2014 International Restless Legs Syndrome Study Group (RLSSG) diagnostic criteria: (1) the urge to move one's extremities due to uncomfortable sensations or pain; (2) the urge starts or worsens during periods of rest or inactivity; (3) the urge is worse in the evening or night; (4) and the urge is partially or totally relieved by movement (6). Notably, the above symptoms are not caused by other medical conditions such as leg cramps, positional discomfort, venous stasis, leg edema or arthritis, and so on. The RLS severity was determined using the validated International Restless Legs Syndrome Severity Scale (IRLS) (20). The LED was calculated according to the established method (21). Epworth Sleepiness Scale (ESS) was used to assess subjective daytime sleepiness and excessive daytime sleepiness (EDS), which was defined as ESS≥10 (22). Depressive symptoms were assessed using the Hamilton Depression Rating Scale−24 (HAMD-24), and patients with a total score equal to or >20 were considered to be experiencing depression (23, 24).

### Polysomnography (PSG)

All recruited patients underwent a standardized, full-night attended, digital video-PSG assessment (Philips Respironics, Murrysville, PA, USA) according to American Academy of Sleep Medicine (AASM) recommendations (25). The PSG recording included standard electroencephalogram (EEG) channels (F3-A2, F4-A1, C3-A2, C4-A1, O1-A2, O2-A1), electrooculogram (EOG), chin and bilateral anterior tibialis electromyogram (EMG), III-lead electrocardiogram (ECG), nasal-oral flow, thoracic and abdominal respiratory efforts, oxygen saturation, and body position. Sleep stages and associated events were manually scored in 30-s blocks according to the criteria described in the American Academy of Sleep Medicine (AASM) manual (25). Poor sleep quality was defined as sleep efficiency (SE) < 80%. OSA was defined as an apnea–hypopnea index (AHI) > 5/h with OSA-related complaints (specified in the ICSD 3rd edition) or >15/h without symptoms (26). Periodic limb movements during sleep (PLMS) were defined as periodic limb movement index (PLMI) > 15 events per hour.

### Statistical Analysis

Descriptive data were presented as means ± standard deviations or frequencies (percentages). Comparisons between two groups for continuous data were conducted using *t*-tests for normally distributed data or the Mann–Whitney *U*-test for non-normally distributed data. Categorical variables were compared using chi-square or the Fisher exact test where appropriate. Spearman's correlation analyses were used to assess the association between objective sleep parameters and potential factors that influenced sleep, including age, PD duration, UPDRS-III, HAMD, PLMI, and AHI. Multiple linear regressions were used to calculate the effects of the potentially related factors on sleep quality in the PD groups with or without RLS. All statistical analyses were conducted using SPSS version 26.0 (SPSS Inc, Chicago, IL, USA). The PSM procedure was performed using the SPSS PSM plug-in “PS Matching.” A *p*-value < 0.05 was considered as statistically significant.

## Results

### Demographic and Clinical Characteristics

In this study, the prevalence of RLS among PD patients was 28.0% (35/125). After screening and PSM, 81 PD patients, including 27 patients with RLS and 54 patients without RLS, were enrolled in this retrospective study. Comparisons of demographic and clinical characteristics between patients with and without RLS are presented in Table 1. There were no significant differences in age at the time of RLS diagnosis, sex, body mass index (BMI), age at onset of PD, and duration of PD between the groups. No differences were observed in the motor phenotype of PD, H-Y stage, LED, HAMD scores, and ESS between PD patients with or without RLS. However, patients with RLS exhibited a higher UPDRS-III score than that of patients without RLS (*p* = 0.001).

**Table 1:** Demographic and clinical characteristics of PD patients with and without RLS.

	**All PD patients**	**PD-RLS**	**PD-NRLS**	***p***
	**(*n* = 81)**	**(*n* = 27)**	**(*n* = 54)**	
Age (years)	62.3 ± 10.0	62.4 ± 9.7	62.3 ± 10.2	0.981
Male (*n*, %)	36 (44.4)	9 (33.3)	27 (50.0)	0.235
BMI (kg/m^2^)	23.3 ± 3.0	22.9 ± 3.0	23.4 ± 3.1	0.500
Age at onset of PD (years)	58.5 ± 9.9	58.1 ± 9.8	58.6 ± 10.1	0.844
Duration of disease (years)	3.7 ± 4.0	4.2 ± 4.3	3.5 ± 3.9	0.427
Motor phenotype (*n*, %)				0.717
Tremor	26 (32.1)	8 (29.6)	18 (33.3)	
Rigidity	37 (45.7)	14 (51.9)	23 (42.6)	
Mixed	18 (22.2)	5 (18.5)	13 (24.1)	
Hoehn and Yahr stage	2.3 ± 0.7	2.4 ± 0.7	2.3 ± 0.7	0.779
UPDRS-III (M)	15.6	18.3	15.6	**0.001**
LED	317.2 ± 180.0	320.8 ± 184.1	315.4 ± 179.6	0.899
HAMD	14.1 ± 5.5	14.7 ± 6.8	13.8 ± 4.7	0.468
Depression (HAMD ≥ 20) (*n*, %)	9 (11.1)	4 (14.8)	5 (9.3)	0.472
ESS	6.0 ± 5.2	6.5 ± 5.1	5.9 ± 5.3	0.598
EDS (ESS ≥ 10) (*n*, %)	25 (30.9)	10 (37.0)	15 (27.8)	0.395
IRLS	—	20.3 ± 6.4	—	—

*PD, Parkinson's disease; RLS, restless legs syndrome; NRLS, without restless legs syndrome; BMI, body mass index; LED, levodopa equivalent dose; UPDRS-III, Unified Parkinson's Disease Rating Scale, Part III; M, the median; HAMD, Hamilton Depression Rating Scale; ESS, Epworth Sleepiness Scale; EDS, excessive daytime sleepiness; IRLS, International Restless Legs Syndrome Severity Scale. A p-value in bold denotes a significant difference (p < 0.05)*.

### Sleep Variables

The comparisons of PSG variables between PD patients with and without RLS are shown in Table 2. PD-RLS patients experienced significantly shorter total sleep times, worse sleep quality, longer REM latency, decreased stage 3 duration, longer wake times after sleep onset, and a higher arousal index than those without RLS. Also, a trend toward an increased proportion of individuals exhibiting difficulty initiating sleep (SL ≥ 30 min) was observed in patients with RLS (40.7 vs. 22.2%, *p* = 0.081). There were no significant differences in sleep latency (SL), duration of sleep stages (S1-3 and REM sleep), AHI, and the proportion of OSA between the two groups. Interestingly, no significant differences were found in PLMI and the proportion of PLMS (PLMI > 15/h) between patients with or without RLS.

**Table 2:** Sleep parameters of PD patients with and without RLS.

	**PD-RLS (*n* = 27)**	**PD-NRLS (*n* = 54)**	***p***
Total sleep time (min)	281.8 ± 89.0	327.7 ± 73.8	**0.016**
Sleep efficiency (%)	60.2 ± 19.1	77.5 ± 59.5	0.145
Poor sleep quality (SE < 0.8) (*n*, %)	17 (47.2)	39 (24.5)	**0.045**
Sleep latency (min)	34.9 ± 48.3	24.7 ± 23.0	0.199
Difficulty initiating sleep (SL ≥ 30 min) (*n*, %)	11 (40.7)	12 (22.2)	0.081
REM latency (min)	219.5 ± 114.4	161.1 ± 98.2	**0.027**
Stage 1 (min)	90.4 ± 41.6	94.5 ± 61.5	0.757
Stage 2 (min)	140.0 ± 65.4	159.2 ± 80.7	0.288
Stage 3 (min)	14.1 ± 16.7	27.3 ± 36.2	**0.027**
Stage REM (min)	37.3 ± 23.0	46.6 ± 35.8	0.160
WASO (min)	139.6 ± 77.5	97.5 ± 68.9	**0.015**
Arousal index (/h)	16.9 ± 12.7	9.2 ± 10.4	**0.005**
PLMI (/h)	19.4 ± 28.5	18.8 ± 30.1	0.932
PLMS (PLMI>15/h) (*n*, %)	10 (37.0)	17 (31.5)	0.617
AHI (/h)	8.9 ± 12.5	8.6 ± 12.6	0.899
OSA (n, %)	13 (48.1)	20 (37.0)	0.337

*PD, Parkinson's disease; RLS, restless legs syndrome; NRLS, without restless legs syndrome; SE, sleep efficiency; SL, sleep latency; REM, rapid eye movement period; WASO, wake after sleep onset; PLMI, periodic limb movement index; PLMS, periodic limb movements during sleep; AHI, apnea–hypopnea index; OSA, obstructive sleep apnea. A p-value in bold denotes a significant difference (p < 0.05)*.

### Correlations Between Sleep Parameters and Clinical Factors

Table 3 shows the correlations between sleep parameters and clinical factors in PD patients with or without RLS. The duration of PD and the UPDRS-III score were significantly negatively correlated with total sleep time (TST) and sleep efficiency in both the two groups. Interestingly, the HAMD score was negatively correlated with TST and sleep efficiency and positively correlated with time awake after sleep onset (WASO) only in PD patients without RLS. PLMI was significantly negatively correlated with TST and sleep efficiency, and positively correlated with SL in PD patients with RLS. However, there were no significant correlations between AHI and sleep parameters including TST, SE, WASO, and SL for both groups.

**Table 3:** Correlations between sleep parameters and clinical factors.

	**Total sleep time (min)**		**Sleep efficiency (%)**		**WASO (min)**		**Sleep latency (min)**	
	**PD-RLS**	**PD-NRLS**	**PD-RLS**	**PD-NRLS**	**PD-RLS**	**PD-NRLS**	**PD-RLS**	**PD-NRLS**
Age	−0.35 (0.07)	–**0.33 (0.02)**	–**0.38 (0.05)**	**0.34 (<0.01)**	0.35 (0.07)	**0.27 (0.05)**	0.29 (0.14)	0.26 (0.06)
PD duration	–**0.50 (<0.01)**	–**0.27 (0.05)**	**−0.50 (0.01)**	**−0.28 (0.04)**	0.37 (0.06)	0.19 (0.17)	0.33 (0.09)	0.01 (0.94)
UPDRS-III	**−0.40 (0.04)**	**−0.37 (<0.01)**	**−0.41 (0.03)**	**−0.38 (< 0.01)**	0.21 (0.29)	**0.34 (<0.01)**	0.16 (0.44)	0.15 (0.28)
HAMD	−0.19 (0.35)	**−0.34 (<** **0.01)**	−0.14 (0.49)	**−0.38 (<** **0.01)**	−0.06 (0.76)	**0.34 (<0.01)**	−0.14 (0.48)	0.11 (0.44)
PLMI	**−0.50 (<0.01)**	−0.26 (0.06)	**−0.53 (<0.01)**	−0.22 (0.11)	0.26 (0.20)	0.15 (0.30)	**0.69 (<0.01)**	0.17 (0.23)
AHI	0.26 (0.19)	−0.26 (0.06)	0.24 (0.23)	−0.26 (0.06)	−0.07 (0.74)	0.15 (0.28)	−0.23 (0.25)	0.16 (0.25)

*WASO, wake after sleep onset; PD, Parkinson's disease; RLS, restless legs syndrome; NRLS, without restless legs syndrome; UPDRS-III, Unified Parkinson's Disease Rating Scale, Part III; HAMD, Hamilton Depression Rating Scale; PLMI, periodic limb movement index; AHI, apnea–hypopnea index. A p-value in bold denotes a significant difference (p < 0.05)*.

### Risk Factors of Sleep Quality

Based on the differences observed in the correlation analysis between the two groups, multiple linear regression was conducted to explore the potential risk factors influencing objective sleep quality in PD patients with or without RLS. Sleep efficiency was considered to be the dependent variable, and various potential factors, including PD duration, UPDRS-III, HAMD, PLMI, and AHI, were independent variables (4, 27, 28). Age and gender were adjusted in the final model.

**Figure 2:**
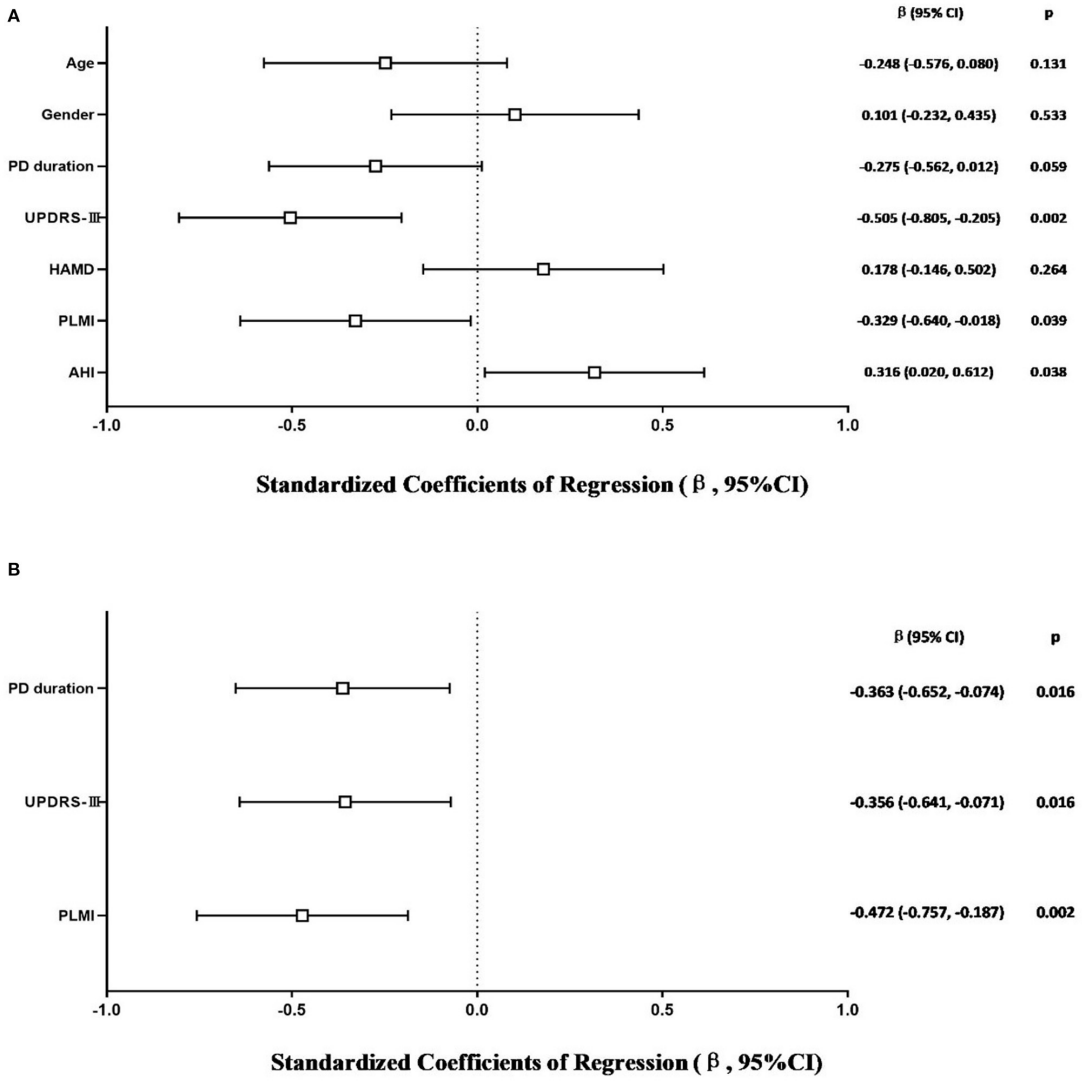
Multiple linear regression analysis of sleep efficiency in PD-RLS patients. The squares are the standardized regression coefficients (β, the change in terms of SDs in the dependent variable that results from a change of 1 SD in an independent variable), and the error bars indicate the 95% CI of β. The gender variant was coded as a dummy variable: male was coded 0, and 1 was used for female. PD, Parkinson's disease; RLS, restless legs syndrome; UPDRS-III, Unified Parkinson Disease Rating Scale Part III; HAMD, Hamilton Depression Rating Scale; PLMI, periodic limb movement index; AHI, apnea–hypopnea index. **(A)** Risk factors of sleep efficiency in PD-RLS patients. All-factors regression (R^2^ = 0.701, *p* = 0.001). **(B)** Stepwise regression (Age- and gender-adjusted, R^2^ = 0.577, *p* < 0.001).

**Figure 3:**
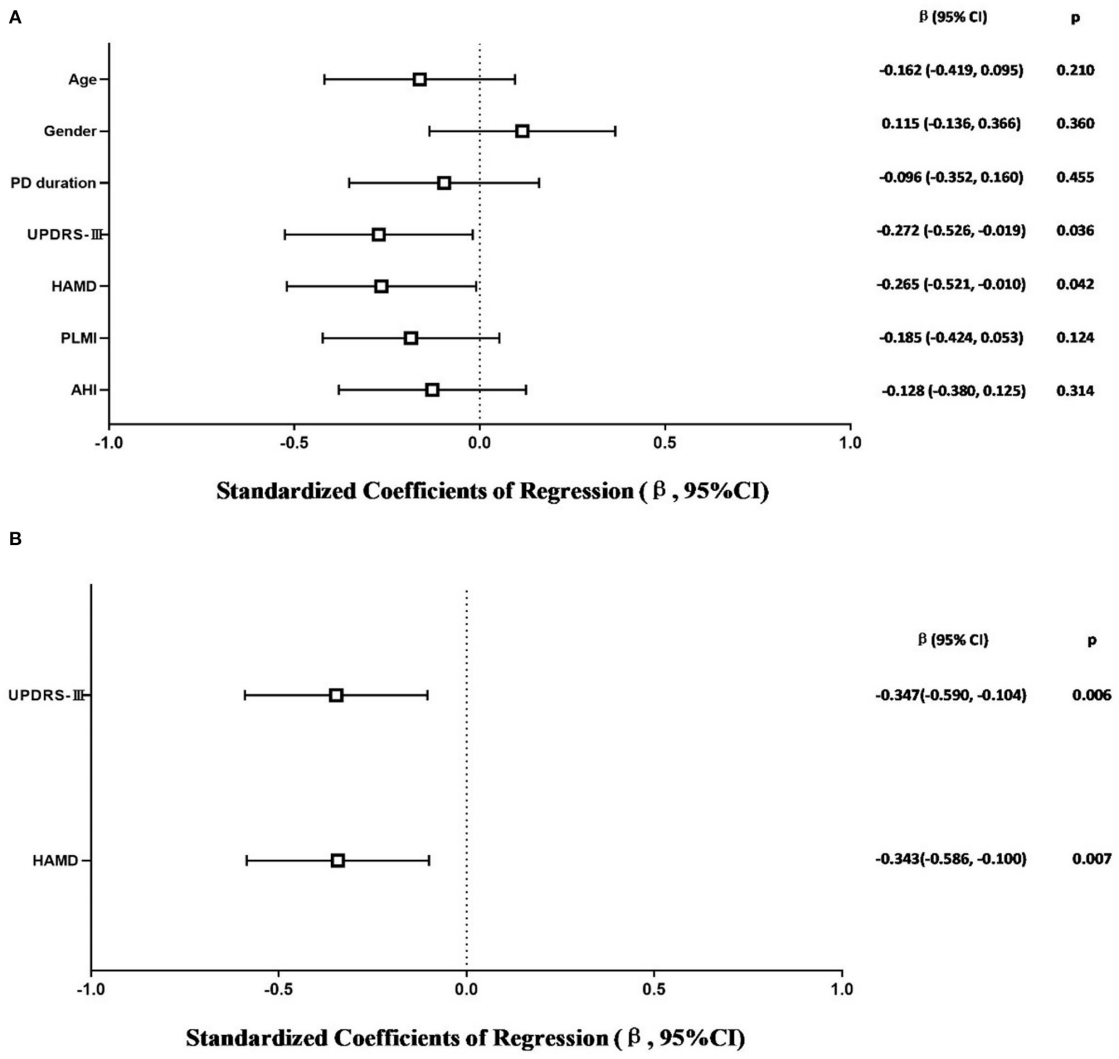
Multiple linear regression analysis of sleep efficiency in PD-NRLS patients. The squares are the standardized regression coefficients (β, the change in terms of SDs in the dependent variable that results from a change of 1 SD in an independent variable), and the error bars indicate the 95% CI of β. The gender variant was coded as a dummy variable: male was coded 0, and 1 was used for female. PD, Parkinson's disease; NRLS, without restless legs syndrome; UPDRS-III, Unified Parkinson Disease Rating Scale Part III; HAMD, Hamilton Depression Rating Scale; PLMI, periodic limb movement index; AHI, apnea–hypopnea index. **(A)** Risk factors of sleep efficiency in PD-NRLS patients. All-factors regression (R^2^ = 0.365, *p* = 0.003). **(B)** Stepwise regression (Age- and gender-adjusted, R^2^ = 0.260, *p* < 0.001).

The model that considered all independent variables explained 76.9% of the variability observed in the sleep efficiency of PD-RLS patients (R^2^ = 0.701, *p* = 0.001). When the independent variables were introduced using a stepwise method, PD duration (β = −0.363, 95% CI: −0.652 to −0.074; *p* = 0.016), UPDRS-III (β = −0.356, 95% CI: −0.641 to −0.071; *p* = 0.016), and PLMI (β = −0.472, 95% CI: −0.757 to −0.187; *p* = 0.002) were the risk factors determined to be associated with sleep efficiency in PD patients with RLS after adjusting for potential confounding variables including age and gender (Figure 2). In PD patients without RLS, the UPDRS-III and HAMD scores were associated significantly with sleep efficiency after adjusting for confounding factors (β = −0.347, 95% CI: −0.590 to −0.104; p = 0.006; β= −0.343, 95% CI: −0.586 to −0.100; *p* = 0.007, respectively) (Figure 3).

## Discussion

To the best of our knowledge, this is the first study to evaluate objective sleep characteristics and potential risk factors of PD patients with and without RLS. After matching the propensity scores of the factors (age and LED) that might have affected sleep quality in PD patients (27, 29), we observed that PD-RLS patients experienced worse nocturnal sleep quality as indicated by a shorter total sleep time, a shorter stage 3 duration, a longer WASO, and a higher arousal index than PD patients without RLS. These results were similar to previous studies that reported worse subjective sleep quality in PD patients with RLS than those without RLS (13, 14). In the current study, we identified several risk factors that influenced sleep quality, including PD duration, UPDRS-III, and PLMI in PD-RLS patients, as well as UPDRS-III and HAMD scores in PD-NRLS patients.

Sleep disorders are complex and diverse in PD patients (30) and are caused by both motor and non-motor symptoms associated with PD. It is well-known that the motor symptoms of PD *per se*, such as difficulty turning over in bed when in the unpredictable “off” state, can result in sleep disturbance associated with nocturnal awakening (31). Unsurprisingly, as a widely used tool to assess the motor performance of PD patients, UPDRS-III was found to be a significant risk factor for sleep quality both in PD-RLS and PD-NRLS patients. Therefore, our findings provided reliable evidence that treatment of sleep disturbance could include improving motor symptoms in PD patients with or without RLS. The UPDRS- III score in PD-RLS patients was significantly higher than that of PD-NRLS patients. Several studies have reported that the diencephalon-spinal pathway, especially abnormalities in the A11 dopaminergic neurons, could lead to RLS (32). Thus, PD-RLS patients might experience a wider range of neurodegeneration and show more severe motor symptoms than PD-NRLS patients.

Depression is one of the most common non-motor symptoms observed in patients with PD and has been found to be associated with worse nighttime sleep in PD (33). In our study, the results revealed that a higher HAMD score was associated with worse objective sleep quality in the PD-NRLS group but not in the PD-RLS group. Similarly, in previous studies, a higher HAMD score was significantly correlated with worse subjective sleep quality of patients with PD as assessed using the PDSS (27, 34).

This current study found that RLS was a common comorbid condition (28.0%) in PD when the International Restless Legs Syndrome Study Group (RLSSG) diagnostic criteria (6) were used. RLS has been reported to be frequent in patients with PD, but whether the prevalence of RLS in PD is higher than in the general population is still a matter of debate (5). The dopaminergic dysfunction is a potential explanation for the high prevalence of RLS in PD. As mentioned, somatic motor neuron activity is known to be mediated through the diencephalon-spinal dopamine pathway, which is proposed to play an important role in the pathophysiology of RLS. A11 neurons might degenerate in patients with PD, and the degeneration of this pathway is believed to be strongly related to the occurrence of RLS in PD patients (35). As is known, patients with RLS primarily report that their sleep disruption is caused by unpleasant sensations and usually report difficulty initiating and maintaining sleep, as well as experiencing unrefreshing sleep two to three times more often than healthy control subjects (36). Also, the objective sleep architecture as assessed by PSG presents with a longer sleep latency and a higher arousal index in RLS. It has been speculated that RLS might share underlying commonalities in pathophysiology with PD (37). Thus, it is noteworthy that differences in motor and non-motor symptoms between PD patients with and without RLS were observed. Currently, some studies have reported that PD patients with RLS exhibit more severe Parkinsonism, worse mental health, and worse sleep quality, using relevant assessment scales (38–40). However, no specific studies have been performed to evaluate sleep in PD patients with and without RLS using objective sleep parameters.

Therefore, this retrospective analysis was conducted on PD patients to evaluate objective sleep architecture and its differences between patients with or without RLS. Concomitant with the subjective studies, the current study observed that PD patients with RLS had worse nocturnal sleep quality compared with PD patients without RLS. In particular, our results demonstrated that PLMI was the risk factor that influenced sleep quality in the PD-RLS group after controlling for potential confounders. Thus, this study provided objective evidence for the effect of RLS on sleep quality in PD patients. Periodic limb movements (PLMs) occur in up to 80% of patients with RLS (41), which can disrupt sleep quantity and quality (42, 43). PLMI is the indicator of PLMS, and our present results suggested that RLS adversely impacted sleep quality in PD patients when PLMI was used as an objective indicator. It has been reported that there is a high prevalence (39–57.8%) of PLMS in PD patients and that reduced striatal dopamine transporter binding and nigrostriatal dopaminergic cell loss might be the underlying reason for the comorbidity of PD and PLMS (44–46). Similarly, in our results, it could explain why there was no significant difference for PLMI between PD-RLS and PD-NRLS patients due to the common pathological mechanism. Notably, in the current study, there was a relatively low occurrence of PLMS in PD-RLS (37%) and PD-NRLS (31.5%) patients, respectively. We speculated that the dopaminergic therapy before PSG assessment in most PD patients could be the important impact factor.

The strengths of the present study include two major points. The first is the objective assessment of sleep quality using PSG. The second is the adjustment of possible factors that influenced sleep quality at the baseline using the PSM model. However, several limitations should be noted in this retrospective study. First, a relatively small sample size could affect the power of the statistical analysis. There were only 27 patients in the PD-RLS group due to the relatively low prevalence of RLS in the study population. Second, the sleep assessment only used PSG, which might result in an overestimation of the sleep quality due to the absence of a subjective sleep evaluation. Third, this study lacked a healthy group to serve as the control. Fourth, we could not evaluate the coexistence of RBD and RLS in patients with PD due to the incomplete data in the diagnosis of RBD. Finally, the sleep architecture could be influenced in part by a “first night effect” due to the collection of data using only a single night data for PSG monitoring. Future studies that include age–sex matched healthy controls are needed to confirm the differences in sleep quality between PD-RLS and PD-NRLS patients and controls.

## Conclusion

This study provided objective evidence that PD-RLS patients have worse nocturnal sleep than PD-NRLS patients. We also observed that several risk factors were associated with the sleep quality in PD patients with or without RLS patients. The finding that PLMS was the risk factor that influenced sleep quality in PD-RLS but not in PD-NRLS patients might suggest that PLMI could be a robust indicator of sleep disruption in PD patients with RLS.

## Data Availability Statement

The raw data supporting the conclusions of this article will be made available by the authors, without undue reservation.

## Ethics Statement

The studies involving human participants were reviewed and approved by Tangdu Ethics Committee and Institutional Review Board. The patients/participants provided their written informed consent to participate in this study.

## Author Contributions

SS implemented the research and collated and analyzed the data. JZ and CS supervised the research. JR responsible for data entry. XZ and JC were responsible for the quality control. All authors read and approved the manuscript.

## Conflict of Interest

The authors declare that the research was conducted in the absence of any commercial or financial relationships that could be construed as a potential conflict of interest.

## References

[1] Zhang ZX Roman GC Hong Z Wu CB Qu QM Huang JB. Parkinson's disease in China: prevalence in Beijing, Xian, and Shanghai. Lancet. (2005) 365:595–7. 10.1016/S0140-6736(05)70801-1 15708103

[2] Muller B Assmus J Herlofson K Larsen JP Tysnes OB. Importance of motor vs. non-motor symptoms for health-related quality of life in early Parkinson's disease. Parkinsonism Relat Disord. (2013) 19:1027–32. 10.1016/j.parkreldis.2013.07.010 23916654

[3] Arnulf I Leu S Oudiette D. Abnormal sleep and sleepiness in parkinson's disease. Curr Opin Neurol. (2008) 21:472–7. 10.1097/WCO.0b013e328305044d 18607209

[4] Zhang Y Ren R Sanford LD Yang L Zhou J Tan L. Sleep in Parkinson's disease: a systematic review and meta-analysis of polysomnographic findings. Sleep Med Rev. (2020) 51:101281. 10.1016/j.smrv.2020.101281 32135452

[5] Stefani A Hogl B. Sleep in Parkinson's disease. Neuropsychopharmacol. (2020) 45:121–28. 10.1038/s41386-019-0448-y PMC6879568 31234200

[6] Allen RP Picchietti DL Garcia-Borreguero D Ondo WG Walters AS Winkelman JW. Restless legs syndrome/Willis–Ekbom disease diagnostic criteria: updated international restless legs syndrome study group (IRLSSG) consensus criteria – history, rationale, description, and significance. Sleep Med. (2014) 15:860–73. 10.1016/j.sleep.2014.03.025 25023924

[7] Yang X Liu B Shen H Li S Zhao Q An R. Prevalence of restless legs syndrome in Parkinson's disease: a systematic review and meta-analysis of observational studies. Sleep Med. (2018) 43:40–6. 10.1016/j.sleep.2017.11.1146 29482811

[8] Ohayon MM O Hara R Vitiello MV. Epidemiology of restless legs syndrome: a synthesis of the literature. Sleep Med Rev. (2012) 16:283–95. 10.1016/j.smrv.2011.05.002 21795081 PMC3204316

[9] Moccia M Erro R Picillo M Santangelo G Spina E Allocca R. A four-year longitudinal study on restless legs syndrome in Parkinson disease. Sleep. (2016) 39:405–12. 10.5665/sleep.5452 26564123 PMC4712388

[10] Poewe W Seppi K Tanner CM Halliday GM Brundin P Volkmann J. Lang: Parkinson disease. Nat Rev Dis Primers. (2017) 3:17013. 10.1038/nrdp.2017.13 28332488

[11] Wijemanne S Jankovic J. Restless legs syndrome: clinical presentation diagnosis and treatment. Sleep Med. (2015) 16:678–90. 10.1016/j.sleep.2015.03.002 25979181

[12] Suzuki K Fujita H Watanabe Y Matsubara T Kadowaki T Sakuramoto H. Leg restlessness preceding the onset of motor symptoms of Parkinson disease: a case series of 5 patients. Medicine. (2019) 98:e16892. 10.1097/MD.0000000000016892 31415433 PMC6831196

[13] Nomura T Inoue Y Miyake M Yasui K Nakashima K. Prevalence and clinical characteristics of restless legs syndrome in Japanese patients with Parkinson's disease. Mov Disord. (2006) 21:380–4. 10.1002/mds.20734 16211604

[14] Gomez-Esteban JC Zarranz JJ Tijero B Velasco F Barcena J Rouco I. Restless legs syndrome in Parkinson's disease. Mov Disord. (2007) 22:1912–6. 10.1002/mds.21624 17579369

[15] Shin HY Youn J Yoon WT Kim JS Cho JW. Restless legs syndrome in Korean patients with drug-naive Parkinson's disease: a nation-wide study. Parkinsonism Relat Disord. (2013) 19:355–8. 10.1016/j.parkreldis.2012.09.009 23047004

[16] Cederberg KL Brinkley EB Belotserkovkaya N Memon RA Motl RW Amara AW. Does restless legs syndrome impact cognitive function via sleep quality in adults with Parkinson's disease? Int J Neurosci. (2020) 130:322–29. 10.1080/00207454.2019.1681423 31625438 PMC7101254

[17] Yu SY Sun L Liu Z Huang XY Zuo LJ Cao CJ. Sleep disorders in Parkinson's disease: clinical features, iron metabolism and related mechanism. PLoS ONE. (2013) 8:e82924. 10.1371/journal.pone.0082924 24376607 PMC3871565

[18] Postuma RB Berg D Stern M Poewe W Olanow CW Oertel W. MDS clinical diagnostic criteria for Parkinson's disease. Mov Disord. (2015) 30:1591–601. 10.1002/mds.26424 26474316

[19] Goetz CG Poewe W Rascol O Sampaio C Stebbins GT Counsell C. Movement disorder society task force report on the hoehn and yahr staging scale: status and recommendations. Mov Disord. (2004) 19:1020–8. 10.1002/mds.20213 15372591

[20] Walters AS LeBrocq C Dhar A Hening W Rosen R Allen RP. Validation of the international restless legs syndrome study group rating scale for restless legs syndrome. Sleep Med. (2003) 4:121–32. 10.1016/S1389-9457(02)00258-7 14592342

[21] Tomlinson CL Stowe R Patel S Rick C Gray R Clarke CE. Systematic review of levodopa dose equivalency reporting in Parkinson's disease. Mov Disord. (2010) 25:2649–53. 10.1002/mds.23429 21069833

[22] Arnulf I Konofal E Merino-Andreu M Houeto JL Mesnage V Welter ML. Parkinson's disease and sleepiness: an integral part of PD. Neurology. (2002) 58:1019–24. 10.1212/WNL.58.7.1019 11940685

[23] Merkl A Schneider GH Schonecker T Aust S Kuhl KP Kupsch A. Antidepressant effects after short-term and chronic stimulation of the subgenual cingulate gyrus in treatment-resistant depression. Exp Neurol. (2013) 249:160–8. 10.1016/j.expneurol.2013.08.017 24012926

[24] Liu X Hou Z Yin Y Xie C Zhang H Zhang H. Dopamine multilocus genetic profile, spontaneous activity of left superior temporal gyrus, and early therapeutic effect in major depressive disorder. Front Psychiatry. (2020) 11:591407. 10.3389/fpsyt.2020.591407 33414733 PMC7782966

[25] AASM. The AASM manual for the scoring of sleep and associated events. In: Rules, Terminology and Technical Specifications. Darien, IL: American Academy of Sleep Medicine (2012).

[26] American Academy of Sleep Medicine (AASM). International Classification of Sleep Disorders (ICSD). 3rd ed. Darien, IL: AASM (2014).

[27] Junho BT Kummer A Cardoso FE Teixeira AL Rocha NP. Sleep quality is associated with the severity of clinical symptoms in Parkinson's disease. Acta Neurol Belg. (2018) 118:85–91. 10.1007/s13760-017-0868-6 29210000

[28] Louter M Munneke M Bloem BR Overeem S. Nocturnal hypokinesia and sleep quality in Parkinson's disease. J Am Geriatr Soc. (2012) 60:1104–8. 10.1111/j.1532-5415.2012.03966.x 22642534

[29] Chang CW Fan JY Chang BL Wu YR. Anxiety and levodopa equivalent daily dose are potential predictors of sleep quality in patients with Parkinson disease in Taiwan. Front Neurol. (2019) 10:340. 10.3389/fneur.2019.00340 31040814 PMC6476952

[30] Comella CL. Sleep disorders in Parkinson's disease: an overview. Mov Disord. (2007) 22(Suppl 17):S367–73. 10.1002/mds.21682 18175398

[31] Kumar S Bhatia M Behari M. Sleep disorders in Parkinson's disease. Mov Disord. (2002) 17:775–81. 10.1002/mds.10167 12210875

[32] Walters AS Rye DB. Review of the relationship of restless legs syndrome and periodic limb movements in sleep to hypertension, heart disease, and stroke. Sleep. (2009) 32:589–97. 10.1093/sleep/32.5.589 19480225 PMC2675893

[33] Zhu K van Hilten JJ Marinus J. Associated and predictive factors of depressive symptoms in patients with Parkinson's disease. J Neurol. (2016) 263:1215–25. 10.1007/s00415-016-8130-3 27126456 PMC4893359

[34] Zhu J Zhong M Yan J Wu Z Pan Y Shen B. Depressive symptoms effect subjective sleep quality in Chinese patients with Parkinson's disease. Clin Neurol Neurosurg. (2020) 195:105950. 10.1016/j.clineuro.2020.105950 32497937

[35] Nomura T Inoue Y Nakashima K. Clinical characteristics of restless legs syndrome in patients with Parkinson's disease. J Neurol Sci. (2006) 250:39–44. 10.1016/j.jns.2006.06.023 16899256

[36] Leschziner G Gringras P. Restless legs syndrome. BMJ. (2012) 344:e3056. 10.1136/bmj.e3056 22623643

[37] Munhoz RP Constantino M Silveira-Moriyama L. The Parkinson's disease and restless legs syndrome/Willis-Ekbom disorder link: evidences, biases and clinical relevance. Arq Neuropsiquiatr. (2019) 77:47–54. 10.1590/0004-282x20180125 30758442

[38] Ylikoski A Martikainen K Partinen M. Parkinson's disease and restless legs syndrome. Eur Neurol. (2015) 73:212–9. 10.1159/000375493 25792198

[39] Fereshtehnejad SM Shafieesabet M Shahidi GA Delbari A Lokk J. Restless legs syndrome in patients with Parkinson's disease: a comparative study on prevalence, clinical characteristics, quality of life and nutritional status. Acta Neurol Scand. (2015) 131:211–8. 10.1111/ane.12307 25263328

[40] Piao Y Lian T Hu Y Zuo L Guo P Yu S. Restless legs syndrome in Parkinson disease: clinical characteristics, abnormal iron metabolism and altered neurotransmitters. Sci Rep. (2017) 7:10547. 10.1038/s41598-017-10593-7 28874701 PMC5585207

[41] Michaud M Paquet J Lavigne G Desautels A Montplaisir J. Sleep laboratory diagnosis of restless legs syndrome. Eur Neurol. (2002) 48:108–13. 10.1159/000062996 12187001

[42] Hornyak M Feige B Voderholzer U Philipsen A Riemann D. Polysomnography findings in patients with restless legs syndrome and in healthy controls: a comparative observational study. Sleep. (2007) 30:861–5. 10.1093/sleep/30.7.861 17682656 PMC1978374

[43] Allen RP Earley CJ. Defining the phenotype of the restless legs syndrome (RLS) using age-of-symptom-onset. Sleep Med. (2000) 1:11–9. 10.1016/S1389-9457(99)00012-X 10733616

[44] Hermann W Flemming T Brandt MD Langner S Reichmann H Storch A. Asymmetry of periodic leg movements in sleep (PLMS) in Parkinson's disease. J Parkinsons Dis. (2020) 10:255–66. 10.3233/JPD-191667 31609696

[45] Covassin N Neikrug AB Liu L Corey-Bloom J Loredo JS Palmer BW. Clinical correlates of periodic limb movements in sleep in Parkinson's disease. J Neurol Sci. (2012) 316:131–6. 10.1016/j.jns.2012.01.004 22277375 PMC3321115

[46] Happe S Pirker W Klosch G Sauter C Zeitlhofer J. Periodic leg movements in patients with Parkinson's disease are associated with reduced striatal dopamine transporter binding. J Neurol. (2003) 250:83–6. 10.1007/s00415-003-0957-8 12527997

